# Comet assay measures of DNA damage as biomarkers of irinotecan response in colorectal cancer in vitro and in vivo

**DOI:** 10.1002/cam4.477

**Published:** 2015-06-23

**Authors:** Joanna P Wood, Andrew J O Smith, Karen J Bowman, Anne L Thomas, George D D Jones

**Affiliations:** Department of Cancer Studies & Molecular Medicine, University of LeicesterLeicester, United Kingdom

**Keywords:** Biomarker, colorectal cancer, comet assay, DNA damage and repair, topoisomerase-I inhibitor

## Abstract

The use of irinotecan to treat metastatic colorectal cancer (CRC) is limited by unpredictable response and variable toxicity; however, no reliable clinical biomarkers are available. Here, we report a study to ascertain whether irinotecan-induced DNA damage measures are suitable/superior biomarkers of irinotecan effect. CRC-cell lines (HCT-116 and HT-29) were treated in vitro with irinotecan and peripheral blood lymphocytes (PBL) were isolated from patients before and after receiving irinotecan-based chemotherapy. Levels of in vitro-, in vivo-, and ex vivo-induced DNA damage were measured using the Comet assay; correlations between damage levels with in vitro cell survival and follow-up clinical data were investigated. Irinotecan-induced DNA damage was detectable in both CRC cell-lines in vitro, with higher levels of immediate and residual damage noted for the more sensitive HT-29 cells. DNA damage was not detected in vivo, but was measurable in PBLs upon mitogenic stimulation prior to ex vivo SN-38 treatment. Results showed that, following corrections for experimental error, those patients whose PBLs demonstrated higher levels of DNA damage following 10 h of SN-38 exposure ex vivo had significantly longer times to progression than those with lower damage levels (median 291 vs. 173 days, *P* = 0.014). To conclude, higher levels of irinotecan-induced initial and residual damage correlated with greater cell kill in vitro and a better clinical response. Consequently, DNA damage measures may represent superior biomarkers of irinotecan effect compared to the more often-studied genetic assays for differential drug metabolism.

## Introduction

Colorectal cancer (CRC) is the second most common cause of cancer-related mortality in the developed world 1,2. Although often curable at a sufficiently early stage, around 20–25% of CRC patients present with metastasis and an additional 25–35% develop metastasis during their illness 3,4. These patients receive systemic treatment with palliative intent, with several licensed cytotoxic and biological agents proven to increase overall survival. First-line combination chemotherapy using 5-Fluorouracil (5-FU) with either oxaliplatin (FOLFOX) or irinotecan (FOLFIRI) has an improved response rate over 5-FU monotherapy alone and is therefore standard first-line treatment 5. Survival is improved if a targeted monoclonal-antibody therapy (anti-VEGFR or anti-EGFR) is added 3,6–10 with VEGF inhibition also being achieved by administering a recombinant fusion protein, namely aflibercept 11 or by inhibition of VEGFR2-TIE2 tyrosine kinase using regorafenib 12. Triplet chemotherapy (FOLFIRINOX) alone or combined with targeted therapies is also a viable option to improve the response rate, however, due to toxicity this regimen is only appropriate in select patient groups 13–15.

The optimal sequence of drug treatment has been the topic for several large prospective phase III studies 5,16–18; the challenge to the clinician being to maximize clinical response but limit toxicities. Generally in terms of efficacy, there is no clearly superior doublet combination regimen 16,19, however, a key point to note is that whilst at a population level the two regimens are comparable, for each individual patient one treatment may be much better in terms of efficacy/tolerability than the other, but currently there is no way of predicting this.

Irinotecan is therefore firmly established as an important drug in the treatment of metastatic CRC. It is a pro-drug initially undergoing hydrolysis to form the active metabolite SN-38 which is 100–1000 times more cytotoxic than the parent drug 20,21. However, irinotecan's metabolism is complex with numerous pathways for deactivation/excretion plus subsequent reactivation both on- and off-target; consequently, high interindividual variation in irinotecan pharmacokinetics and response exists. The unpredictable pharmacokinetics alongside the narrow therapeutic window of irinotecan may lead to overtreatment, with unacceptable toxicities arising in approximately one-third of patients receiving this drug 22–26. Conversely, some patients may be undertreated so receiving a suboptimal therapeutic effect. Irinotecan is currently prescribed, using a patient's body surface area, at doses derived from clinical trials based on outcomes across populations; this approach does not account for interindividual differences.

The need for a predictive test of irinotecan response and/or toxicities is well recognized; illustrated by a plethora of literature articles detailing attempts to develop such a test. Much of this research has involved testing for tumor somatic mutations and germline changes which may be used to identify pharmacogenetic variations in drug metabolism and so predict irinotecan response/toxicity; however, these studies have generally not been validated and have not altered clinical practice 27. *UGT1A1* is the most widely investigated gene to date. An increased number of TA repeats in the TATA box in its promoter region (wild type *n* = 6) has been shown to correlate with reduced enzyme expression leading to lower glucuronidation rates and thus higher levels of, and prolonged exposure to, SN-38 28,29. In 2007, a meta-analysis of nine studies concluded that the risk of hematological toxicities was increased in patients homozygous for the UGT1A1*28 polymorphism (defined by the presence of 7 TA repeats) at medium or high doses of irinotecan treatment (>150 mg/m^2^) 30. However, the FOCUS study (the largest CRC randomized control trial to assess candidate pharmocogenetic markers to date) did not show a significant association of UGT1A1*28 with toxicity in patients receiving either irinotecan monotherapy or the FOLFIRI combination 31. Thus, routine testing for this polymorphism has not been adopted worldwide owing to the presence of conflicting negative data and lack of endorsement by specialist societies 32. Similarly, studies of polymorphisms of other candidate genes including: CES, CYP3A, other UGT genes, membrane transporter and DNA repair genes have failed to yield a robust biomarker 31,33–38.

A key weakness of these previous studies is that they failed to account for the entire collective effects of the enzymes, transporters and environmental factors, both known and unknown, that are involved in this drug's metabolism; at least half of which has been shown to be unexplained by genotype 34. This study was therefore undertaken with the aim of investigating a superior method to predict toxicities and response to irinotecan chemotherapy. It was proposed that a study of the mechanism of action of this drug, rather than focusing on its metabolism, may yield more clinically useful findings. Irinotecan is a topoisomerase I (topo-I) inhibitor that exerts its cytotoxic effect by causing DNA damage. SN-38 induces single-stranded DNA breaks (SSBs) by stabilizing the complex formed by topo-I and DNA 39–42. These SSBs then generate toxic double-stranded breaks (DSBs) by replication fork collapse and ultimately trigger apoptosis 43. This leads to the proposed research hypothesis that “DNA damage is a biomarker of irinotecan effect.” This hypothesis was based on reports that irinotecan kills cancer cells by inducing DNA damage and that the toxicities of irinotecan are due to the overaccumulation of damaging SN-38 off-target 44. Measures of DNA damage are readily achieved in cancer cells in vitro and on easily accessible normal cells, for example, peripheral blood lymphocytes (PBLs), in vivo by the Comet assay. As DNA damage is the key endpoint of irinotecan's effects, one could speculate that it would be a strong surrogate marker for of all of the factors affecting SN-38 metabolism and it's binding to topo-I. Thus, if this hypothesis was proven to be true, it would indicate an advantage in delivering a predictive test based on DNA damage over methods already researched.

In this study, we report the combined findings of an investigation to ascertain whether DNA damage, as assessed by alkaline Comet assay (ACA), induced following irinotecan exposure is predictive of cancer cell response in vitro, plus the design and conduct of the first prospective clinical study to assess whether DNA damage induced in PBLs following irinotecan or SN-38 exposure are potential predicitve biomarkers of drug effect.

## Materials and Methods

### Chemicals

Chemicals and cell culture reagents were obtained from Sigma-Aldrich Company Ltd., Poole, Dorset, UK unless otherwise stated.

### Cell lines and culture conditions

HCT-116 and HT-29 cell lines were obtained from American Type Culture Collection (ATCC), Manassas, VA. HCT-116 were grown in Dubecco's modified eagle's medium with 4500 mg glucose/L, 110 mg sodium pyruvate/L and l-glutamine, plus 10% fetal calf serum (FCS) (Invitrogen, Paisley, UK). HT-29 were grown in McCoy's 5A + GlutaMAX-1 (Invitrogen), plus 10% FCS. Both lines were grown at 37°C in 5% CO_2_.

### Irinotecan treatment of cell lines

Cells were plated at densities of 200,000 cells per well on plastic 6 well tissue culture plates (except controls which were plated at 50,000 cells per well) and left at 37°C to attach. Irinotecan solutions were prepared in appropriate volumes of culture medium; adjusting the final Dimethyl sulfoxide (DMSO) concentration to 0.3%; the control solution contained 0.3% DMSO. Cells were incubated with irinotecan solutions of 0, 1, 5, 10, 15, and 20 *μ*mol/L for 3, 8, 24, 48, and 72 h at 37°C, washed free of the drug, harvested, counted, and frozen in culture medium containing 5% DMSO prior to Comet analysis.

### Clonogenic survival assay

Cells were plated at densities of 400 cells per plastic Petri dish, left at 37°C to attach and then treated for 24 h with irinotecan solutions of 0, 1, 3, 10, 30, 100, 300, and 1000 nmol/L, washed free of the drug and incubated in culture medium at 37°C until the formation of visible colonies.

### Ethics statement

The clinical study was approved by Nottingham Research Ethics Committee 1 (reference number 09/H0403/8). Trial participants were identified as those aged over 18 years who were due to receive irinotecan-based chemotherapy for metastatic CRC at Leicester Royal Infirmary. Written informed consent was obtained from each patient before study entry.

### Patients and blood samples

Initially, 3 × 10 mL blood samples were collected from each patient in heparinized vials (Sarstedt, Nümbrecht, Germany) before, 1 h after and 24 h after treatment. Following an interim analysis, a substantial amendment was made to the trial protocol so that only 1 × 20 mL blood sample was obtained prior to chemotherapy.

### PBL isolation and culture

Samples were coded, kept at room temperature and processed as quickly as possible following venepuncture. Blood was mixed with an equal volume of RPMI 1640 media and PBLs isolated using density centrifugation with Ficoll-paque™ PLUS (GE Healthcare, Chalfont St Giles, Buckinghamshire, UK). A proportion of cells from the prechemotherapy sample were seeded at a density of 2.5–5 × 10^5^/mL in a minimum volume of 10 mL and cultured in Quantum 724 complete media for primary lymphocyte culture (QBL; PAA Laboratories Ltd., Yeovil, Somerset, UK) for 72 h prior to treatment to assess ex vivo damage. The remaining cells were stored in RPMI plus 20% FCS and 10% DMSO at -80°C prior to analysis of DNA damage induced in vivo.

### PBL treatments

Stock solutions of irinotecan and SN-38 were prepared in DMSO and stored at −20°C. The ex vivo treatments were undertaken by serial dilutions of the stock solutions in QBL media. Optimization assays were performed over a range of doses and time points; 0–100 *μ*mol/L (irinotecan) and 0–10 *μ*mol/L (SN-38) for 1–12 h. For the ex vivo clinical study PBLs were treated for 1 h with 0, 0.01, 0.1, 0.5, 1.0, 2.5, and 5 *μ*mol/L SN-38 and for 4 and 10 h with 5 *μ*mol/L SN-38. Following treatment the cells were centrifuged at 300*g* for 5 min at 4°C and then processed using the following assays.

### Alkaline Comet assay

Thawed frozen PBLs or those gathered immediately following treatments were used for this assay depending whether drug exposure occurred in vivo or ex vivo, respectively. Samples from each patient were processed simultaneously, in triplicate, in a single-electrophoresis tank alongside three HT-29 cell controls (a DMSO only negative control and two positive controls treated with either 1 *μ*mol/L SN-38 for 1 h or 10 Gy irradiation).

The assay was a modified version of that described by Olive et al. 45. PBLs embedded on slides in 0.6% low melting point agarose gels were lysed overnight then incubated in electrophoresis buffer for 20 min before being electrophoresed for 20 min (0.6 V/cm). They were then neutralized and propidium iodide (PI) stained prior to imaging and analysis using Komet analysis software version 5.5 (Andor technology, Belfast, UK). A total of 300 cells were analyzed per sample (50 per each of two gels on triplicate slides). The mean and standard error of the median percentage tail DNA from these triplicates were calculated.

Both raw and corrected results were analyzed. A correction factor was determined by dividing the average difference in % tail DNA of all controls by the difference for each individual experiment. The sample results from each experiment were then multiplied by the correction factor.

### Genotyping of the *UGT1A1* variable length (TA)n repeat polymorphism (UGT1A1*28)

Genomic DNA was prepared from frozen PBLs using standard techniques (Qiagen, Manchester, UK). Polymerase chain reaction was performed to amplify a 254-bp region of the *UGT1A1* gene using 5′-TATCTCTGAAAGTGAACTC-3′ sense and 5′-ATCAACAGTATCTTCCCAG-3′ antisense primers 36. Sequencing was performed by the PNACL facilities at the University of Leicester using Big-Dye Version 3.1 chemistry on an Applied Biosystems, Paisley, UK model 3730 automated capillary DNA sequencer.

### Data analysis

Clinical data were obtained by reviewing the patients’ notes. Toxicities were graded according to the Common Toxicity Criteria (CTC) Version 4 (2009). Best response was assessed using the Response Evaluation Criteria In Solid Tumors (RECIST) criteria version 1.0. Statistical analysis was performed using either PASW statistics 18.0 for Windows or SPSS 14.0 for Windows SPS Inc., Chicago, IL, 5 September 2005). For the Comet assay data one-way analysis of variance (ANOVA) test was performed with post hoc Tukey's test. Elsewhere, *P* values were calculated using the independent samples *t*-test or the Chi-squared test for trend. *P* values are significant at <0.05.

## Results

### In vitro studies

#### Cell survival and proliferation

**Figure 1:**
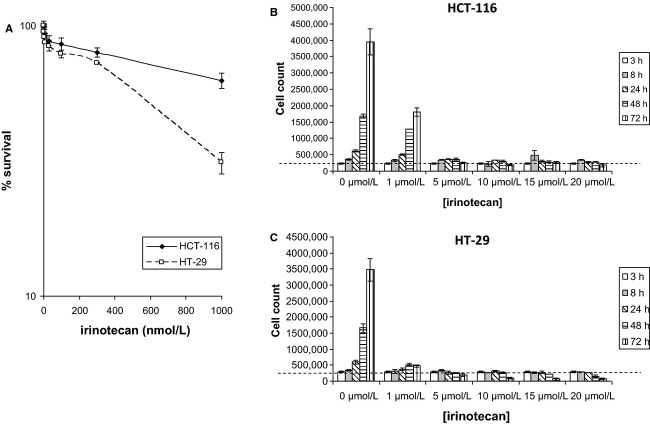
In vitro cell survival and proliferation data. (A) Cell survival curves for HCT-116 and HT-29 cells treated with irinotecan doses of 0, 1, 3, 10, 30, 100, 300, and 1000 nmol/L (±SE of the mean of three independent experiments) over 24 h as determined by clonogenic survival assay. (B) HCT-116 and (C) HT-29 cell counts (±SE of mean of three independent experiments) following 3, 8, 24, 48, and 72 h of irinotecan treatment at concentrations of 0, 1, 5, 10, 15, and 20 *μ*mol/L. Broken line represents initial numbers of cells seeded – 200,000 cells per well.

The cytotoxic and antiproliferative effects of irinotecan on HCT-116 and HT-29 cells were determined by clonogenic survival and cell counting assays. Figure1A shows clonogenic cell survival curves following a 24-h treatment with irinotecan at doses of 1-1000 nmol/L; the data reveal the HT-29 cells to be more chemo-sensitive than HCT-116 cells. Figure1B and C depict cell counting assays for HCT-116 and HT-29 cells, respectively, following treatment with irinotecan at doses of between 1 and 20 *μ*mol/L for time periods of between 3 and 72 h; the cell counts were compared to the initial number of cells seeded (as denoted by the dashed line) to allow an estimation of cytotoxicity. A substantial inhibitory effect of irinotecan on cell proliferation of both cells was observed, with the HT-29 cells showing heightened sensitivity toward the drug. Figure1B also reveals that at no time point did the cell count for the HCT-116 cells fall consistently below the initial number of cells seeded; in contrast, irinotecan was clearly shown to be cytotoxic to HT-29 cells following the 72-h treatments with the 5, 10, 15, and 20 *μ*mol/L doses and following the 48-h treatments with the 15 and 20 *μ*mol/L doses. Therefore, measures of clonogenic survival match the measures of cytotoxicity with the HT-29 cells being shown more sensitive to the cell killing effects of irinotecan.

#### DNA damage formation and repair

**Figure 2:**
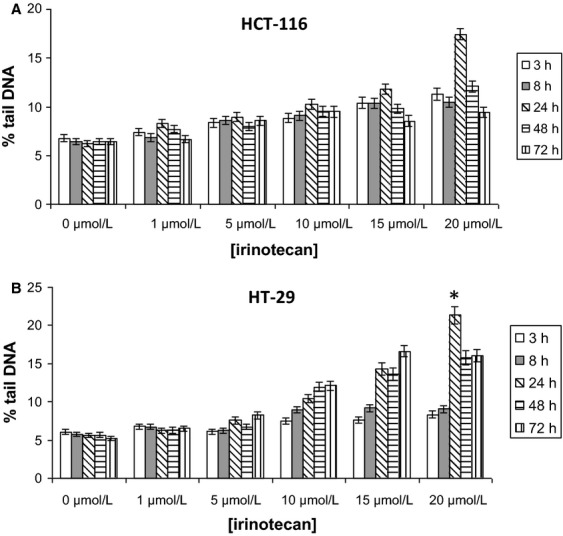
In vitro DNA damage and repair data. (A) HCT-116 and (B) HT-29 irinotecan dose response. Cells were treated with irinotecan doses of 0, 1, 5, 10, 15, and 20 *μ*mol/L for time periods of 3, 8, 24, 48, and 72 h. DNA damage was measured as percentage tail DNA ± SE of the mean of data pooled together from of three independent experiments. *Denotes the HT-29 cells having significantly higher levels of induced damage compared to the HT-116 cells (*P* = 0.003) following 24-h treatment with 20 *μ*mol/L irinotecan.

Treatment of HCT-116 and HT-29 cells with irinotecan at doses of 5–20 *μ*mol/L for 3, 8, and 24 h indicated a consistent dose–response relationship (Fig.2A and B). The 24-h treatment at 20 *μ*mol/L produced the highest measures of damage in both cell lines with the HT-29 cells having significantly higher levels of induced damage compared to the HT-116 cells (*P* < 0.005). Following 48 and 72 h of treatment with 20 *μ*mol/L irinotecan, the percentage tail DNA was lower than at 24 h for both cell lines, suggesting the repair of irinotecan-induced DNA-stranded breaks. However, the levels of residual DNA damage were clearly greater in the HT-29 cells (Fig.2B) compared to the HT-116 cells (Fig.2A). Therefore, HT-29 cells are shown to be both more damage sensitive and demonstrate higher levels of residual damage; both these findings agree with the data from both the clonogenic survival (Fig.1A) and cell counting (Fig.1B and C) assays.

### In vivo/ex vivo studies

The in vitro data indicates that assessment of DNA damage formation and repair in biopsied target CRC tumor cells might be a good predicative measure of CRC tumor response to irinotecan. However, having access to such target tissue is not usually possible and so a surrogate target tissue was sought for the in vivo studies. Lymphocytes are considered to be a good surrogate tissue (possessing host characteristics) and are frequently used in studies where target tissue is not readily attained 46; consequently, patient PBLs were studied in vivo and ex vivo.

#### Patient demographics and treatment

Forty-two patients were recruited. Blood samples were obtained prior to the first cycle of chemotherapy in 22 patients; the remainder was obtained prior to subsequent cycles. At the conclusion of clinical data collection 37 patients had died, four were still alive and one had been lost to follow-up. Following detection of disease progression, over a fifth of the participants received further systemic cancer treatment.

Only one patient was treated with irinotecan monotherapy (350 mg/m^2^), all others received combination regimens (39 had FOLFIRI at a starting irinotecan dose of 180 mg/m^2^, one received FOLFIRI at 135 mg/m^2^, and one capecitabine/irinotecan 250 mg/m^2^). Two of the patients receiving FOLFIRI also received bevacizumab and a further 12 had their treatment combined with either an oral endothelin receptor antagonist (ZD4054) or a placebo as part of the FOLFERA study 47.

The general demographics and baseline characteristics of all trial participants and of individuals grouped according to the subsequent development of grade 3/4 toxicities and response to treatment are summarized in Table1. Patient characteristics were well matched within both the toxicity and response subgroups with the only exception being that those with toxicities were significantly more likely to have a poorer performance status (PS) than those who tolerated treatment well (*P* = 0.017, calculated using the Chi-squared test for trend).

**Table 1:** Baseline characteristics of all clinical trial participants and the corresponding data when patients were grouped according to the development of grade 3/4 toxicities (diarrhea and neutropenia) and response to treatment

		Toxicity groups		Response groups	
	All patients	≤ Grade 2 toxicities	Grade 3–4 toxicities	Clinical benefit (PR/SD)	Progressive disease
Number of assessable patients	42 (100)	31 (74)	11 (26)	29^2^	7^2^
Sex					
Male	27 (64)	20 (65)	7 (64)	19 (66)	4 (57)
Female	15 (36)	11 (35)	4 (36)	10 (34)	3 (43)
Median age (range)	64 (34–77)	62 (34–77)	67 (61–74)	62 (44–76)	68 (34–77)
Race					
Caucasian	39 (93)	28 (91)	11 (100)	26 (90)	7 (100)
Asian	2 (5)	2 (6)	0	2 (7)	0
Afro-Caribbean	1 (2)	1 (3)	0	1 (3)	0
ECOG PS at baseline					
0	17 (40)	16 (52)	1 (9)^1^	15 (52)	1 (14)
1	23 (55)	14 (45)	9 (82)	13 (45)	6 (86)
2	2 (5)	1 (3)	1 (9)	1 (3)	0
Status of primary					
Resected	17 (40)	10 (32)	7 (64)	11 (38)	2 (29)
Unresected	22 (52)	19 (61)	3 (27)	16 (55)	5 (71)
Local recurrence	3 (7)	2 (6)	1 (9)	2 (7)	0
Site of metastasis					
Locally advanced	3 (7)	3 (10)	0	3 (10)	0
Liver	5 (12)	4 (13)	1 (9)	2 (7)	2 (29)
Liver + others	23 (55)	19 (61)	4 (36)	17 (59)	4 (57)
None liver	11 (26)	5 (16)	6 (5)	7 (24)	1 (14)
Metastatectomy peri-irinotecan					
Yes	3 (7)	2 (6)	1 (9)	2 (7)	0
No	39 (93)	29 (94)	10 (91)	27 (93)	7 (100)
UGT1A1^*^1^*^1	21 (50)^3^	14 (45)	7 (64)	13 (45)	4 (57)
UGT1A1^*^1^*^28	15 (36)	12 (39)	3 (27)	11 (38)	3 (43)
UGT1A1^*^28^*^28	6 (14)	5 (16)	1 (9)	5 (17)	0

Values within parenthesis are expressed in percentage.

1 Statistically significant with *P* < 0.05 calculated using the chi-squared test for trend.

2 Six patients did not have response assessed due to either the absence of measurable disease or the premature cessation of treatment as a result of toxicities or death.

3 These gene frequencies were in Hardy–Weinberg equilibrium (*P* = 0.50 calculated using the chi-squared test).

There were no significant associations of UGT1A1*28 homozygotes with either toxicities or response to treatment although it was observed that all assessable patients with this genotype had at least stabilization of disease (Table1).

The median time to progression (TTP) was 217 days (assessable in 35 patients) and median OS was 320 days. There were no significant differences in TTP or OS between those with toxicities and those who tolerated treatment well, and similarly there were no significant associations with *UGT1A1* status. As expected, those who progressed on treatment had inferior survival (median OS 191 vs. 397 days, *P* = 0.001).

#### In vivo study

**Figure 3:**
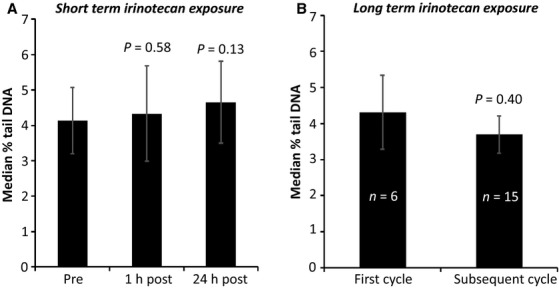
In vivo study results. Bar graphs illustrating the cumulative results of the DNA damage measured in PBLs isolated (A) before and shortly after irinotecan treatment and (B) prior to receiving the first or subsequent cycles of irinotecan chemotherapy. Results are an average of the median percentage tail DNA across 21 assessable patients’ samples. *P* values were calculated using the independent samples *t*-test compared to baseline.

This study was undertaken to determine if irinotecan treatment in vivo leads to an increase in PBL DNA damage, as detected by ACA, and was performed on samples obtained from the first 21 patients recruited to the clinical study. The DNA damage levels across all clinical samples were minimal compared to those of the irradiated controls that were processed in parallel (mean percentage tail DNA 4.36% vs. 17.5%). Collectively, there was no significant difference in the mean percentage tail DNA either 1 h or 24 h post irinotecan treatment compared to pretreatment baseline (Fig.3A). The ACA was also unable to detect evidence of an effect of long-term irinotecan exposure as illustrated by the observation that there was no difference in background DNA damage levels for patients prior to receiving their first cycle of treatment compared to those due to receive subsequent cycles (Fig.3B). Therefore, following an interim analysis demonstrating these negative results, this in vivo part of the clinical study was terminated.

#### Detecting DNA damage in PBLs treated with irinotecan or SN-38 ex vivo

**Figure 4:**
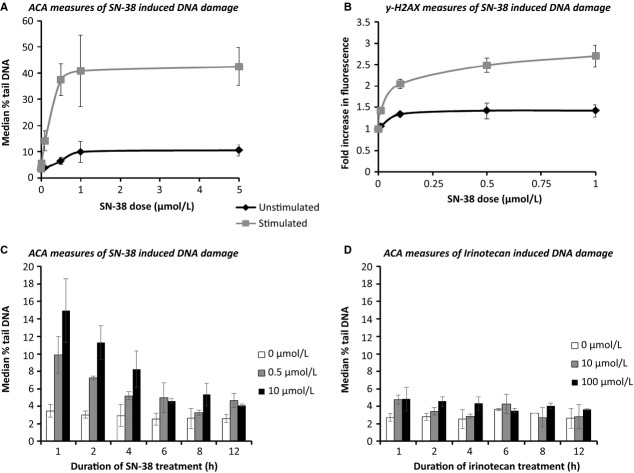
Optimization of the ex vivo study assays. DNA damage measured using (A) ACA and (B) ƴ-H2AX detection in PBLs cultured in the presence or absence of a mitogen, prior to treatment with SN-38 for 1 h and DNA damage detected over a 12-h time course in PBLs cultured with PHA stimulation for 72 h prior to treatment with (C) irinotecan and (D) SN-38.

A series of laboratory experiments were next performed in order to investigate the negative in vivo study results and also to determine whether conditions could be established to enable irinotecan to induce measurable DNA damage ex vivo. Only minimal DNA damage was induced in unmanipulated (unstimulated) PBLs treated with SN-38 (Fig.4A) ex vivo. It was postulated that since these cells usually reside in the nonreplicating G_0_ phase of the cell cycle 48 they may not possess sufficient topo-I to mediate SN-38-induced SSB formation. Additionally, if not progressing through S phase, then the replication fork would not advance and the subsequent toxic DSBs not formed. Cell cycle analysis was thus performed and confirmed that the proportion of PBLs in S phase increased from <20% to >50% by on mitogenic stimulation with phytohaemagglutinin (PHA) (see Fig. S1).

For PBLs cultured with PHA stimulation for 72 h prior to SN-38 exposure, significant levels of DNA strand break damage were induced and detected by ACA (Fig.4A) and measurement of *γ*-H2AX (Fig.4B). The response was maximal following 1 h of exposure and reduced over time, with the active metabolite SN-38 (Fig.4C) producing a far greater response than the prodrug irinotecan (Fig.4D). These initial data were used to establish a method to proceed with the ex vivo component of the clinical study.

#### Ex vivo study

This study was undertaken to determine if SN-38 treatment ex vivo leads to an increase in PBL DNA damage, as detected by ACA and measurement of *γ*-H2AX, and was performed on samples obtained from 40 of the trial participants. With ACA, a dose response was detected in all patients as illustrated by an initial increase in DNA damage with rising SN-38 dose followed by a plateau at the higher doses when the response became saturated (full data are provided in Table S1 with a representative dose–response curve illustrated in Fig.4A). Results showed a wide range of interindividual variation in the level of DNA damage detected; correlations of both raw and corrected laboratory results with clinical data were investigated as described below.

The maximum % tail DNA (range 6.35–54.23%) detected in each patient did not correlate with clinical outcome or genotype. Similarly, the gradient of the initial dose–response curve (between 0 and 0.5 *μ*mol/L) and percentage tail DNA detected at subphysiological, physiological, and supraphysiological doses were all individually investigated but once again, when patients were classified according to either UGT1A1*28 status, toxicities or response to chemotherapy, no significant differences in DNA damage between these groups were detected. Additionally, there were no significant associations of the raw DNA damage data at any dose with TTP or OS.

The absolute maximum DNA damage measured in samples from each individual was detected at the highest (5 *μ*mol/L) treatment dose of SN-38 used in 27 (68%) of the patients. The remainder had maximum damage detected following exposure to lower doses and demonstrated plateauing of the dose–response curve (6 at 2.5 *μ*mol/L, 6 at 1 *μ*mol/L, and 1 at 0.5 *μ*mol/L). There was no significant correlation of the dose of ACA response saturation with *UGT1A1* status or with toxicities to treatment, however, none of the patients with progressive disease (PD) exhibited a plateau at doses lower than 5 *μ*mol/L illustrating a possible, albeit not statistically significant, association with clinical response (*P* = 0.075, calculated using the Chi-squared test for trend) (see Fig. S2). Although this test had 100% sensitivity to detect patients with PD, its positive predictive value (PPV) was only 27% thus limiting any potential clinical utility.

DNA damage was maximal at 1 h, reducing over time (ca. 10 h) in 37 patients. Two patients, one of whom experienced severe toxicities had maximum damage occurring at 4 h and one patient, also experiencing severe toxicities, had maximum damage at 10 h, but there were no significant correlations of the raw time course data with the clinical outcomes.

**Figure 5:**
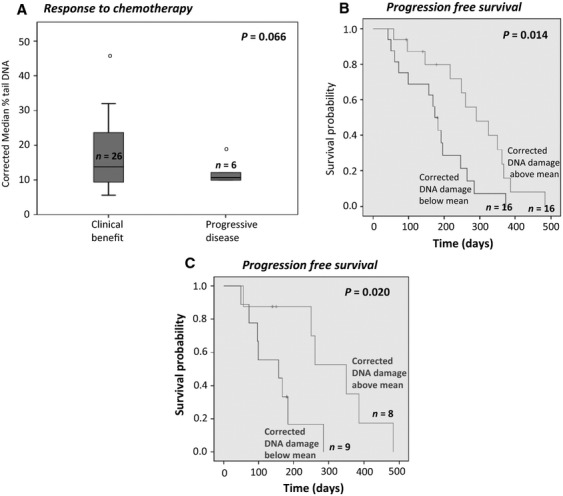
Ex vivo study results. Correlating corrected ACA data with clinical outcome. (A) is a box and whisker plot demonstrating the association of response to irinotecan chemotherapy with corrected DNA damage induced in PBLs treated with 0.5 *μ*mol/L SN-38 for 1 h. (B) is a Kaplan–Meier plot demonstrating the TTP for patients grouped according to the level of corrected DNA damage, induced in PBLs treated with 5 *μ*mol/L SN-38 for 10 h. (C) is a Kaplan-Meier plot demonstrating the TTP for patients selected according to irradiated control being within 1 standard deviation of the mean grouped according to level of DNA damage adjusted using the selected irradiated control correction factor, measured using the ACA, induced in PBLs treated *ex vivo* with: 5 μM SN-38 for 4 hours. *P* values were calculated using the log rank test.

To assess whether experimental error was masking any clinical associations, control data were also analyzed. The presence of interexperimental variation was confirmed; DNA damage in the irradiated control cells was more consistent than in those control cells treated with SN-38 (coefficient of variation 0.25 vs. 0.54). The more consistent irradiated controls (results available in 37 patients) were therefore used to correct the raw ex vivo data. There was no association of this corrected data with toxicities to treatment or *UGT1A1* genotype. In 32 assessable patients, it was observed that SN-38 induced DNA damage following 1 h treatment was generally lower in those with PD but this did not reach statistical significance (Fig.5A). However, it was noted that TTP was significantly increased in those patients with higher corrected DNA damage at 10 h of drug exposure (median 291 vs. 173 days, *P* = 0.014) (Fig.5B). This was further supported by the observation that TTP was also significantly increased in selected patients with higher corrected DNA damage following 4 h of drug exposure; these subjects being selected according to their irradiated control being within 1 standard deviation of the mean and grouped according to level of DNA damage adjusted using the selected irradiated control correction factor. This latter analysis was undertaken in an attempt to assess whether trends could be strengthened if the assay variability was less and, on this basis, 22 patients were selected to have similar assay efficacy. Clearly this analysis was limited due to smaller patient numbers but, within this selected group, six had severe toxicities, four had PD, and two were UGT1A1*28 homozygotes.

## Discussion

Individualization of irinotecan chemotherapy using robust evidence-based prediction of efficacy and toxicity is a highly sought goal. Indeed, in this study 40% of patients experienced grade 3/4 toxicities (*n* = 11) and/or had a best response of PD (*n* = 7) and thus would have benefitted from a predictive biomarker of irinotecan's effects.

When designing this study, it was initially proposed that Comet measures of irinotecan induced-DNA damage levels would represent an ideal mechanistic biomarker of drug effect and that measuring the extent of treatment-induced DNA damage in whole cells would take into account many of the important molecular, genetic and epigenetic circumstances that will ultimately dictate/influence a cell's response to treatment. The advantage of this approach of using the Comet assay to measure drug-induced cellular DNA damage is that intact cells can express both protein systems involved in drug activation processes and also the various detoxification pathways/cellular defense mechanisms. The extent of drug-induced DNA damage levels therefore represents the balance between these two processes and may more accurately reflect treatment sensitivity in patients. While predicting response to therapy based on analysis of single-molecular markers remains an attractive proposition, this approach is probably too simplistic and “all-inclusive” cell-based procedures such as the Comet assay represent a realistic way forward.

This study has demonstrated that higher levels of irinotecan-induced initial and residual DNA damage, as assessed by ACA, correlated with both greater CRC cell kill in vitro and a better clinical response in vivo, and consequently that laboratory measures of DNA damage may permit the prediction of response and prognosis in patients with metastatic CRC receiving this drug. This would aid the identification of those who may not benefit and so could be spared exposure and consequent unnecessary toxicities from this treatment. However, the results have also shown that these measures of DNA damage in PBLs are not predictive of irinotecan toxicities and thus do not have the potential to personalize the dose administered.

A potential weakness in this protocol was that by treating the PBLs with SN-38, the opportunity to detect any interindividual variation due to differences in the metabolism of the irinotecan pro-drug was lost. However, as the majority of toxicities are thought to be due to the slow glucuronidation of SN-38 44,49, it was decided that the higher DNA damage levels induced using this metabolite would be more informative and more likely to detect interindividual differences than the lower levels detected following irinotecan exposure.

Evidence that DNA damage may be a potential predictive biomarker of irinotecan response in vivo was chiefly provided by the observation that those individuals with high levels of DNA damage after 10 h of SN-38 treatment ex vivo had a significantly longer TTP. It was also noteworthy that following analysis of selected corrected data that the TTP was significantly longer in those having higher DNA damage levels following 4 h of irinotecan exposure.

Further evidence that DNA damage may be a biomarker to predict irinotecan response was also provided by the finding that none of the patients with PD exhibited saturation of the dose–response curve at doses lower than 5 *μ*mol/L SN-38, whereas the response plateaued in some of those with clinical benefit. It is plausible that the requirement of a high dose of SN-38 to detect a plateau in the laboratory response could be indicative of resistance to treatment and with increased patient numbers this result may have achieved significance. Although this finding had 100% sensitivity to detect patients with PD, a poor PPV limited this finding; this could potentially be improved by a more detailed assessment of the 2.5–5 *μ*mol/L SN-38 dose range thus enabling more accurate identification of the plateau dose within this range.

The TTP data from this research demonstrate a probable role of DNA repair in resistance to treatment, thus highlighting the importance of further investigating specific DNA repair gene activity as potential biomarkers, although the largest biomarker study in metastatic CRC conducted to date did not show any predictive value of the two DNA repair genes studied (XRCC1 and MLH1) with irinotecan outcome 31.

The lack of correlation of DNA damage with toxicities was likely to be due, at least in part to the fact that PBLs were not an optimal normal tissue surrogate in which to investigate irinotecan effect. Indeed, LC-MS/MS analysis (see Figs. S2 and S3) confirmed that PBLs hydrolyzed irinotecan only weakly (Fig. S4) and did not perform SN-38 glucuoronidation ex vivo (Figs. S5 and S6); this may also account for the lack of association of homozygosity for UGT1A1*28 with DNA damage. It is possible that DNA damage induced in an alternative normal tissue surrogate, with a metabolism more closely resembling that occurring in vivo, would be a superior biomarker-model for toxicities. As hydrolysis of irinotecan and glucuronidation of SN-38 occur primarily in the liver, hepatic tissue would thus be the most likely to yield positive results, but the risks and discomfort of performing a liver biopsy would likely outweigh any potential clinical benefit. Ongoing genotyping work to develop a predictive panel of genes is more likely to successfully deliver a clinically acceptable test for toxicities.

Whilst the observation that measures of DNA damage in PBLs correlate with tumor response (TTP) needs to be interpreted with caution, due to the relatively small patient numbers and the need to apply a correction factor, it does generate indications for future work. The study of irinotecan/SN-38 induced DNA damage on target CRC cells to predict response is ideally warranted. The clinical utility of studying CRC cells obtained from biopsies would be limited due to the risks involved in obtaining a fresh biopsy. However, if CRC cultures could be obtained using a minimally invasive procedure, for example, from circulating tumor cells, then the assessment of whether laboratory measures of irinotecan/SN-38 induced DNA damage correlates with response to treatment would be justified. In this setting, the Comet assay as a potential predictive test has distinct advantages. It is straightforward, rapid, has a low material requirement, is relatively cheap and it can be automated; all features that make it suitable for routine testing in a clinical context. The assay has been successfully used to demonstrate that treatment sensitivity can be measured in a range of tumor cell lines (reviewed by McKenna et al. 50).

In conclusion, higher levels of irinotecan-induced DNA damage correlated with greater cell kill in vitro and with measures of a beneficial clinical response in vivo. Consequently, laboratory measures of DNA damage and repair may represent superior, more versatile biomarkers of irinotecan's effect compared to genetic assays for differential drug metabolism. Further studies of DNA damage as predictive biomarkers of tu mor response are warranted.

## Supporting Information

Table S1. Ex vivo study results: raw data demonstrating the dose response of PBLs treated with SN-38 for 1 h assessed using the ACA. **Table S2.** The dose of assay saturation in all trial participants and those grouped according to UGT1A1 status, toxicities and response to irinotecan treatment. **Figure S1.** Cell cycle analysis of PBLs cultured (A) without mitogenic stimulation in RPMI media and (B) with mitogenic PHA stimulation in QBL media. **Figure S2.** MS analysis of (A) SN-38 and (B) irinotecan standards. **Figure S3.** Typical LC-MS/MS SRM ions chromatograms for (A) the SN-38 standard (10 pmol) and (B) the irinotecan standard (10 pmol). **Figure S4.** Typical LC-MS/MS SRM ions chromatograms for (A) solvent blank (20 mmol/L ammonium acetate pH 3.5/acetonitrile [80:20, v/v]) (B) PBL cells treated ex vivo with DMSO (control) and (C) PBL cells treated ex vivo with 50 *μ*mol/L irinotecan for 4 h. **Figure S5.** Typical LC-MS/MS SRM ions chromatograms for PBL cell extracts from donor 1 treated with (A) DMSO control (B) 0.05 *μ*mol/L SN-38 for 4 h and (C) 5 *μ*mol/L SN-38 for 4 h. **Figure S6.** Typical LC-MS/MS SRM ions chromatograms for PBLs cell extracts from donor 2 treated with 5 *μ*mol/L SN-38 for (A) 6 h and (B) 24 h.
