# Supplementary material for: Comet assay measures of DNA damage as biomarkers of irinotecan response in colorectal cancer in vitro and in vivo
Source: Cancer Med. 2015 Jun 23;4(9):1309–21. doi: 10.1002/cam4.477 (PMC4567016; doi:10.1002/cam4.477)
Supplement: Table S1. — Ex vivo study results: raw data demonstrating the dose response of PBLs treated with SN-38 for 1 h assessed using the ACA. Table S2. The dose of assay saturation in all trial participants and those grouped according to UGT1A1 status, toxicities and response to irinotecan treatment. Figure S1. Cell cycle analysis of PBLs cultured (A) without mitogenic stimulation in RPMI media and (B) with mitogenic PHA stimulation in QBL media. Figure S2. MS analysis of (A) SN-38 and (B) irinotecan standards. Figure S3. Typical LC-MS/MS SRM ions chromatograms for (A) the SN-38 standard (10 pmol) and (B) the irinotecan standard (10 pmol). Figure S4. Typical LC-MS/MS SRM ions chromatograms for (A) solvent blank (20 mmol/L ammonium acetate pH 3.5/acetonitrile [80:20, v/v]) (B) PBL cells treated ex vivo with DMSO (control) and (C) PBL cells treated ex vivo with 50 μmol/L irinotecan for 4 h. Figure S5. Typical LC-MS/MS SRM ions chromatograms for PBL cell extracts from donor 1 treated with (A) DMSO control (B) 0.05 μmol/L SN-38 for 4 h and (C) 5 μmol/L SN-38 for 4 h. Figure S6. Typical LC-MS/MS SRM ions chromatograms for PBLs cell extracts from donor 2 treated with 5 μmol/L SN-38 for (A) 6 h and (B) 24 h. [file cam40004-1309-sd1.docx]

**Supporting Information: for Review and Publication On-Line**

**Table SI-1. *Ex vivo* study results: Raw data demonstrating the dose response of PBLs treated with SN-38 for 1 hour assessed using the ACA**

| Patient ID | Treatment regimen & cycle sampled | Median % tail DNA *(s.d.)* | | | | | | | | | |
| --- | --- | --- | --- | --- | --- | --- | --- | --- | --- | --- | --- |
|  |  | 0 μM SN-38 | 0.01 μM SN-38 | 0.1 μM SN-38 | 0.5 μM SN-38 | 1.0 μM SN-38 | 2.5 μM SN-38 | 5.0 μM SN-38 | HT-29 negative control | HT- 29 SN-38 control | HT-29 irradiated control |
| 001 | FOLFIRI 7 | n/a | n/a | n/a | n/a | n/a | n/a | n/a | n/a | n/a | N/A |
| **002** | **FOLFIRI 4** | **n/a** | **n/a** | **n/a** | **n/a** | **n/a** | **n/a** | **n/a** | **n/a** | **n/a** | **N/A** |
| **003** | **FOLFIRI 11** | **3.71**  ***(0.63)*** | **3.98**  ***(0.79)*** | **5.28**  ***(1.81)*** | **14.86**  ***(0.81)*** | **15.36**  ***(1.35)*** | **18.82**  ***(1.33)*** | **N/A** | **3.80**  ***(0.57)*** | **13.18 *(2.01)*** | **N/A** |
| 004 | FOLFIRI 2 | 6.98  *(1.45)* | 7.94  *(0.92)* | 10.32  *(2.69)* | 12.5  *(2.54)* | 18.73  *(3.85)* | 16.83  *(2.78)* | N/A | 6.05  *(0.97)* | 26.47  *(6.91)* | N/A |
| 005 | FOLFIRI 2 | 4.13  *(1.28)* | 6.59  *(2.91)* | 4.72  *(0.46)* | 8.01  *(1.46)* | 25.95 *(19.30)* | 24.27 *(15.33)* | 10.94  *(2.02)* | 5.22  *(4.22)* | 13.40  *(12.93)* | 17.03  *(14.95)* |
| 006 | FOLFIRI 2 | 6.6  *(2.12)* | 7.86  *(0.41)* | 11.92  *(0.76)* | 20.74  *(3.31)* | 19.69  *(3.79)* | 18.81  *(2.25)* | 18.91  *(4.42)* | 5.65  *(1.08)* | 37.70  *(2.88)* | 14.42  *(1.63)* |
| 007 | FOLFIRI 10 | 4.06  *(1.50)* | 5.37  *(0.42)* | 10.04  *(2.34)* | 22.56  *(1.87)* | 23.27  *(3.66)* | 23.06  *(2.77)* | 25.25  *(0.53)* | 4.58  *(1.44)* | 24.38  *(3.35)* | 18.50  *(2.54)* |
| 008 | FOLFIRI 6 | 5.11  *(0.19)* | 5.23  *(0.76)* | 15.13  *(0.04)* | 27.91  *(3.22)* | 33.10  *(1.72)* | 32.24  *(2.12)* | 38.63  *(2.41)* | 4.39  *(0.34)* | 25.95  *(0.69)* | 16.64  *(1.84)* |
| 009 | FOLFIRI 1 | 4.30  *(0.29)* | 4.63  *(1.30)* | 9.24  *(1.61)* | 15.06  *(1.38)* | 17.06  *(3.35)* | 23.13  (*1.83)* | 25.45  *(3.26)* | 4.24  *(1.11)* | 18.20  *(2.13)* | 12.91  *(0.62)* |
| 010 | FOLFIRI 3 | 6.24  *(1.34)* | 7.03  *(2.21)* | 9.13  *(0.98)* | 12.36  *(2.51)* | 13.59  *(3.99)* | 19.77  *(6.31)* | 20.23  *(5.35)* | 4.74  *(1.23)* | 70.68  *(8.73)* | 16.76  *(4.96)* |
| **011** | **FOLFIRI 1** | **6.33**  ***(1.31)*** | **7.62**  ***(1.01)*** | **12.27**  ***(2.09)*** | **21.72**  ***(4.18)*** | **20.49**  ***(1.30)*** | **22.49**  ***(2.45)*** | **25.65**  ***(3.38)*** | **3.62**  ***(1.13)*** | **22.24**  ***(2.98)*** | **22.53**  ***(3.88)*** |
| **012** | **FOLFIRI 4** | **7.80**  ***(1.32)*** | **9.52**  ***(1.65)*** | **14.46**  ***(3.29)*** | **18.80**  ***(0.06)*** | **18.96**  ***(0.47)*** | **19.03**  ***(2.86)*** | **22.29**  ***(2.16)*** | **2.97**  ***(1.13)*** | **31.16**  ***(0.77)*** | **15.52**  ***(0.94)*** |
| 013 | FOLFIRI 2 | 5.39  *(1.54)* | 6.20  *(1.40)* | *17.93*  *(1.76)* | 20.16  *(3.09)* | 16.25  *(1.59)* | 24.94  *(2.98)* | 24.99  *(2.43)* | 3.65  *(0.62)* | 19.97  *(1.26)* | 17.62  *(0.62)* |
| 014  *^28^ | FOLFIRI 3 | 3.93  *(0.32)* | 4.23  *(0.66)* | 6.31  *(1.05)* | 13.10  *(2.16)* | 14.20  *(1.45)* | 17.62  *(2.08)* | 21.74  *(1.97)* | 3.05  *(0.63)* | 28.76  *(4.42)* | 16.78  *(1.40)* |
| 015 | FOLFIRI 2 | 6.70  *(0.65)* | 6.94  *(2.39)* | 7.11  *(4.07)* | 8.51  *(1.65)* | 11.38  *(1.28)* | 16.54  *(2.93)* | 13.06  *(2.02)* | 3.94  *(0.84)* | 30.39  (5.86) | 16.24  *(0.63)* |
| 016 | FOLFIRI 1 | 6.47  *(1.12)* | 5.90  *(1.28)* | 7.71  *(1.64)* | 12.05  *(1.43)* | 16.06  *(0.35)* | 15.81  *(3.09)* | 18.49  *(0.89)* | 3.86  *(1.39)* | 21.66  *(4.16)* | 19.02  *(2.06)* |
| 017 | FOLFIRI 1 | 5.51  *(2.65)* | 5.35  *(1.04)* | 8.66  *(0.29)* | 19.26  *(4.00)* | 27.68 *(11.61)* | 27.72  *(9.92)* | 31.46  *(8.89)* | 4.87  *(2.03)* | 17.48  *(4.87)* | 23.88  *(6.79)* |
| 018  *^28^ | FOLFIRI 2 | 4.26  *(0.49)* | 5.00  *(0.77)* | 9.20  *(1.26)* | 14.97  *(1.33)* | 23.11  *(5.03)* | 17.66  *(3.63)* | 21.21  *(1.80)* | 4.13  *(0.42)* | 23.18  *(6.21)* | 22.47  *(4.08)* |
| 019 | FOLFIRI 1 | 3.02  *(0.93)* | 4.19  *(1.73)* | 9.41  *(0.73)* | 22.01  *(3.36)* | 24.65  *(2.29)* | 26.99  *(0.32)* | 31.15  *(5.28)* | 3.38  *(0.69)* | 31.25  *(2.64)* | 20.60  *(3.95)* |
| 020  *^28^ | FOLFIRI 1 | 4.21  *(0.53)* | 4.42  *(1.15)* | 6.84  *(1.37)* | 12.30  *(0.75)* | 14.50  *(2.10)* | 20.44  *(1.34)* | 20.76  (*0.88)* | 3.58  *(1.15)* | 20.69  *(1.38)* | 23.99  *(3.55)* |
| 021 | FOLFIRI 2 | 3.67  *(0.52)* | 4.85  *(0.93)* | 11.86  *(1.76)* | 16.34  *(3.62)* | 23.42  *(4.13)* | 23.28  *(2.30)* | 27.57  *(3.72)* | 2.67  *(0.53)* | 17.89  *(2.84)* | 21.02  *(4.19)* |
| 022 | FOLFIRI 1 | 3.92  *(1.90)* | 3.50  *(0.39)* | 6.76  *(2.00)* | 9.18  *(1.51)* | 10.47  *(1.67)* | 12.27  *(1.51)* | 12.64  *(1.93)* | 3.64  *(1.51)* | 24.07  *(1.61)* | 20.80  *(2.42)* |
| 023  *^28^ | FOLFERA3 | 3.04  *(0.82)* | 3.23  *(0.55)* | 7.78  *(0.62)* | 10.30  *(0.62)* | 12.75  *(2.50)* | 16.35  *(2.50)* | 15.47  *(1.59)* | 2.14  *(0.72)* | 13.85  *(0.53)* | 15.87  *(1.47)* |
| **024** | **FOLFERA 1** | **4.11**  ***(0.15)*** | **4.03**  ***(0.99)*** | **6.39**  ***(1.34)*** | **10.63**  ***(1.86)*** | **12.66**  ***(2.95)*** | **13.87**  ***(2.03)*** | **14.07**  ***(0.58)*** | **3.65**  ***(1.68)*** | **20.27**  ***(2.34)*** | **20.05**  ***(1.71)*** |
| 025 | FOLFERA 1 | 4.30  *(0.78)* | 4.56  *(0.64)* | 7.51  *(3.21)* | 16.95  *(6.50)* | 18.43  *(5.42)* | 21.25  *(9.03)* | 22.88  *(8.88)* | 4.04  *(0.84)* | 25.32  *(8.70)* | 26.37  *(8.50)* |
| 026 | FOLFERA 1 | 4.66  *(0.39)* | 6.68  *(2.18)* | 9.99  *(0.48)* | 15.44  *(0.86)* | 18.41  *(0.16)* | 18.92  *(1.97)* | 20.76  *(3.27)* | 3.45  *(0.20)* | 17.87  *(5.00)* | 25.51  *(7.71)* |
| 027 | Irinotecan1 | 3.46  *(1.08)* | 3.96  *(0.38)* | 6.03  *(1.11)* | 12.09  *(2.16)* | 17.93  *(1.19)* | 18.62  *(1.47)* | 17.52  *(2.69)* | 2.97  *(0.55)* | 36.22  *(1.70)* | 22.45  *(1.60)* |
| **028** | **FOLFERA 1** | **2.97**  ***(0.15)*** | **3.62**  ***(0.97)*** | **12.38**  ***(3.58)*** | **20.31**  ***(2.42)*** | **31.06**  ***(5.53)*** | **36.80**  ***(5.31)*** | **38.30**  ***(7.83)*** | **2.34**  ***(0.55)*** | **15.53**  ***(3.35)*** | **15.65**  ***(3.02)*** |
| 029 | FOLFERA 3 | 5.63  (0.93) | 6.17  *(2.02)* | 10.68  *(2.04)* | 16.16  *(2.87)* | 19.58  *(0.36)* | 22.36  *(2.01)* | 19.66  *(2.69)* | 4.12  *(1.10)* | 12.48  *(1.23)* | 13.23  *(0.63)* |
| **030** | **FOLFERA 1** | **5.28**  ***(1.04)*** | **5.28**  ***(0.65)*** | **7.93**  ***(1.11)*** | **12.36**  ***(0.74)*** | **12.30**  ***(3.67)*** | **16.78**  ***(1.02)*** | **22.02**  ***(1.35)*** | **3.46**  ***(1.32)*** | **12.50**  ***(2.79)*** | **14.49**  ***(2.09)*** |
| 031 | FOLFERA 1 | 4.40  *(0.27)* | 4.60  *(0.68)* | 5.91  *(0.56)* | 5.60  *(1.23)* | 8.33  *(1.15)* | 9.51  *(2.38)* | 10.27  *(2.84)* | 4.63  *(0.89)* | 11.20  *(1.93)* | 18.24  *(4.92)* |
| **032** | **FOLFERA 1** | **4.55**  ***(1.13)*** | **4.51**  ***(1.10)*** | **4.33**  ***(0.24)*** | **5.75**  ***(0.55)*** | **6.66**  ***(1.34)*** | **6.07**  ***(1.19)*** | **7.77**  ***(1.75)*** | **3.82**  ***(0.66)*** | **11.36**  ***(1.21)*** | **13.21**  ***(1.36)*** |
| 033 | FOLFERA 2 | 4.65  *(0.85)* | 3.85  *(0.56)* | 5.56  *(1.13)* | 6.56  *(1.82)* | 8.17  *(2.70)* | 7.16  *(0.52)* | 8.65  *(0.85)* | 3.85  *(0.73)* | 10.68  *(1.21)* | 12.17  *(0.28)* |
| 034 | FOLFIRI  2 | 4.09  *(0.85)* | 4.99  *(0.90)* | 5.15  *(1.77)* | 7.14  *(0.89)* | 10.20  *(1.44)* | 12.37  *(1.05)* | 13.87  *(4.10)* | 3.70  *(0.93)* | 13.59  *(0.74)* | 10.59  *(2.41)* |
| **035** | **FOLFIRI**  **1** | **4.19**  ***(1.23)*** | **3.94**  ***(0.42)*** | **3.87**  ***(0.60)*** | **5.74**  ***(1.88)*** | **4.25**  ***(0.53)*** | **6.35**  ***(0.25)*** | **5.72**  ***(0.45)*** | **3.05**  ***(0.30)*** | **10.80**  ***(2.84)*** | **8.89**  ***(1.04)*** |
| **036**  *^28^ | **Cap/iri**  **1** | **3.46**  ***(0.24)*** | **3.56**  ***(0.24)*** | **4.59**  ***(0.40)*** | **5.48**  ***(1.79)*** | **7.33**  ***(0.61)*** | **9.28**  ***(1.10)*** | **9.49**  ***(0.94)*** | **3.47**  **(0.45)** | **10.74**  **(0.84)** | **11.40**  **(1.29)** |
| 037 | FOLFIRI 1 | 4.04  *(0.82)* | 4.99  *(0.64)* | 8.26  *(1.50)* | 11.10  *(3.98)* | 14.26  *(3.30)* | 12.71  *(0.82)* | 15.66  *(4.93)* | 3.40  *(1.21)* | 21.56  *(7.85)* | 18.86  *(4.56)* |
| 038 | FOLFIRI/  avastin 1 | 3.81  *(0.91)* | 4.91  *(0.48)* | 6.44  *(1.10)* | 10.28  *(0.87)* | 11.22  *(1.93)* | 13.45  *(1.79)* | 13.79  *(2.15)* | 3.25  *(0.36)* | 15.11  *(0.20)* | 17.28  *(1.66)* |
| 039 | FOLFIRI/  avastin 3 | 4.49  *(0.83)* | 5.96  *(0.82)* | 8.55  *(1.15)* | 10.32  *(1.72)* | 11.93  *(0.76)* | 13.80  *(1.31)* | 15.07  *(2.55)* | 3.85  *(0.20)* | 15.23  *(1.80)* | 17.96  *(3.17)* |
| 040 | FOLFERA 1 | 3.81  *(0.52)* | 6.81  *(2.13)* | 17.80  *(5.16)* | 35.24 *(10.47)* | 38.65  *(6.50)* | 54.23 (*16.64)* | 49.62 *(10.64)* | 4.44  *(0.54)* | 50.74  *(13.31)* | 14.76  *(1.00)* |
| **041** | **FOLFERA 1** | **5.48**  ***(1.21)*** | **5.24**  ***(0.72)*** | **11.94**  ***(0.49)*** | **18.53**  ***(2.43)*** | **23.30**  ***(5.42)*** | **20.18**  ***(3.31)*** | **22.44**  ***(4.05)*** | **3.68**  ***(1.40)*** | **19.22**  ***(3.39)*** | **12.90**  ***(1.22)*** |
| 042  *^28^ | FOLFIRI 1 | 4.94  *(1.81)* | 6.74  *(1.93)* | 12.02  *(2.17)* | 16.83  *(1.09)* | 20.96  *(1.24)* | 20.63  *(4.21)* | 18.89  *(3.21)* | 3.80  *(1.03)* | 10.46  *(1.36)* | 12.09  *(1.49)* |

s.d is standard deviation from 3 samples within the same electrophoresis tank, N/A is result /sample not available.

Those in **bold** font experienced grade 3/4 toxicities (diarrhoea or neutropenia). Those highlighted best response was progressive disease

^*28^ = UGT1A1*28 homozygote.

**Table SI-2. The dose of assay saturation in all trial participants and those grouped according to UGT1A1 status, toxicities and response to irinotecan treatment**

| **Dose of ACA saturation**  **(μM SN-38)** | **All patients** | **Toxicity groups** | | **Response groups** | | **UGT1A1 *28 groups** | |
| --- | --- | --- | --- | --- | --- | --- | --- |
|  |  | **≤ Grade 2 toxicities** | **Grade 3/4 toxicities** | **Clinical benefit (PR/SD)** | **PD** | **≥1 wt allele present** | **UGT1A1 *28*28** |
| 0.5 | 1 (2%) | 1 (3%) | 0 | 1 (4%) | 0 | 1 (3%) | 0 |
| 1 | 6 (15%) | 5 (17%) | 1 (10%) | 6 (21%) | 0 | 4 (12%) | 2 (33%) |
| 2.5 | 6 (15%) | 4 (13%) | 2 (20%) | 5 (18%) | 0 | 5 (15%) | 1 (17%) |
| 5 | 27 (68%) | 20 (67%) | 7 (70%) ^T^ | 16 (57%) | 6 ^R^ | 24 (70%) | 3 (50%) ^U^ |

NB/ T, R and U represent p values calculated using the Chi-Squared test for trend. T= 0.590, R=0.075 and U=0.333

**Figure SI-1. Cell cycle analysis of PBLs cultured A) without mitogenic stimulation in RPMI media and B) with mitogenic PHA stimulation in QBL media.** Following isolation, PBLs were resuspended in Quantum 724 complete media for primary lymphocyte culture (QBL). They were seeded at a density of 2.5 - 5 x 10^5^ /ml in 25 cm^2^ flasks and cultured for 72 hours prior to being used for experiments. The flasks were gently agitated every 24 hours. PBLs were confirmed to cycle using this QBL media (B). In addition, the cell cycle analysis of cells cultured in RPMI media (unstimulated) had the presence of a sub G1 peak implying that apoptosis was occurring under these suboptimal conditions (A).

**Liquid chromatography-mass spectrometry (LC-MS)**

The LC-MS system consisted of a Waters Alliance 2695 separations module with a 100 μL injection loop connected to a Micromass Quattro Ultima Pt. (Waters Ltd, Manchester, UK) tandem quadrupole mass spectrometer with an electrospray ionisation interface. The temperature of the electrospray source was maintained at 110 °C and the desolvation temperature at 350 °C. Nitrogen gas was used as the desolvation gas (650 L/h) and the cone gas (25 L/h). The capillary voltage was set at 3.00 kV. The cone and RF1 lens voltages were 40 V and 60 V, respectively. The photomultiplier was set at 850 V. The mass spectrometer was tuned by using a standard solution of SN-38 which was diluted to a concentration of 100 pmol/μL using 0.1% formic acid/acetonitrile (75:25, v/v) from a stock solution dissolved in DMSO and introduced by continuous infusion at a flow rate of 10 μL/min with a Harvard model 22 syringe pump (Havard Apparatus Ltd., Edenbridge, UK). A 10 μL aliquot of each sample or standard was injected on to a Hypersil C18 HyPurity, 15 cm x 2.1 mm, 3 μm analytical column plus guard column (1 cm x 2.1 mm, 3 μm) and 102 Krudkatcher filter (0.5 μm). The column was located in the column oven and maintained at 35 °C. The mobile phase consisted of solvent A, 0.1% formic acid and solvent B, acetonitrile. The flow rate was 0.12 mL/min and the following gradient was used to elute the column; 0 min- 22% B, 5min -22% B, 15 min – 45% B, 20 min – 65% B., 20.1 min – 22 % B and 35 min – 22% B. The samples were analysed in positive ionization tandem mass spectrometry (MS/MS) selected reaction monitoring (SRM) mode for the [M+H]+ ion to major fragment transitions of irinotecan *m/z* 587 to 543, SN-38 *m/z* 393 to 349 and SN-38 G *m/z* 569 to 393. The collision gas was argon (indicated cell pressure 2.0 × 10-3 mbar) and the collision energy set at 21 eV. The dwell time was set to 200 ms and the resolution was 1.5 *m/z* units at peak base. The data was processed using MassLynx software (version 4.1, Waters Ltd.).

**Figure SI-2. MS analysis of A) SN-38 and B) irinotecan standards.** Since standards for the SN-38G was not available, the mass spectrometer tuning parameters were optimised using the authentic SN-38 and irinotecan standards. SN-38 and irinotecan standards were diluted with 0.1% formic acid/acetonitrile (75:25, v/v) and full scan MS spectrum and the MS/MS product ion spectrum recorded as described above. Full scan MS analysis of the SN-38 standard showed the presence of an ion at *m/z* 393.46 corresponding to the protonated molecule ion ([M+H]^+^) expected for SN-38. Following MS/MS analysis of the SN-38 standard the major product ion formed from the SN-38 [M+H]^+^ precursor ion (*m/z* 393) was at *m/z* 349 corresponding to the loss of –CO_2_ (A). Similarly for the irinotecan standard, full scan MS analysis showed the presence of an ion at *m/z* 587.38 corresponding to the protonated molecule ion ([M+H]^+^) expected. Following MS/MS analysis, the major product ions formed from the irinotecan [M+H]^+^ precursor ion (*m/z* 587) were at *m/z 124, 195, 167, 110, 543 and 502* (B).

**Figure SI-3. Typical LC-MS/MS SRM ions chromatograms for A) the SN-38 standard (10 pmol) and B) the irinotecan standard (10 pmol).** The SRM mode transitions used for the detection of SN-38 and irinotecan were [M+H]^+^ *m/z* 393 to 349 and M+H]^+^ *m/z* 587 to 543, respectively. The unavailability of a SN-38G standard meant that the SRM mode transition used for its detection, which was [M+H]^+^*m/z* 569 to 393, was obtained from the literature (Santos *et al.* *Clin Cancer Res, 6, 2012-20*). The typical LC-MS/MS SRM ions chromatograms for the detection of SN-38 and irinotecan standards are shown in A and B. Irinotecan and SN-38 eluted with retention times of 7.5 and 15.7 minutes respectively. It should be noted that a relatively small peak was observed at 7.5 minutes for the irinotecan standard in the SN-38 SRM channel which may be attributable to in source fragmentation of the irinotecan, and in addition a small amount of SN-38 carry over was detected at 15.64 minutes (B).

**Figure SI-4. LC-MS/MS SRM analysis of PBLs treated with irinotecan *ex vivo*.** The typical LC-MS/MS SRM ions chromatograms obtained for the analysis of PBLs treated with irinotecan *ex vivo* are shown. A relatively small peak corresponding to SN-38 was detected at 15.55 min following the *ex vivo* exposure of PBLs to 50 μM ironotecan for 4 hours indicating that PBLs have the capacity to metabolise irinotecan *ex vivo* (C). To confirm that the SN-38 peak detected was not due to carryover, a series of solvent blank injections were performed prior to injection of the test sample for which no peaks were observed (A). It should be noted that a relatively small peak was observed at 7.7 min corresponding to irinotecan in PBLs exposed to DMSO *ex vivo* which may be attributable to contamination of the DMSO (B).

**Figure SI-4. Typical LC-MS/MS SRM ions chromatograms for A) solvent blank (20mM ammonium acetate pH 3.5/acetonitrile (80:20, v/v)) B) PBL cells treated *ex vivo* with DMSO (control) and C) PBL cells treated *ex vivo* with 50 μM irinotecan for 4 hours**

**Figure SI-4 continued**

**Figure SI-5 & SI-6. Analysis of the glucuorinidation capacity of PBLs following treatment with SN-38 *ex* vivo.** Two different SN-38 dosing treatments were used to investigate the ability of PBLs to produce glucuronide metabolites. A low dose (0.05 μM) reflecting the physiological dose and a higher dose (5 μM) were used to treat the PBLs. The typical LC-MS/MS SRM ions chromatograms for the detection of SN-38 in the low and high dose treatments for 4 hours are shown in Figures SI-5 B and SI-5 C. SN-38 was clearly detectable in the 5 μM treated samples, however for the 0.05 μM treated cells the size of the SN-38 peak detected was greatly reduced (Figure SI-5 B shown by the arrow) and is at the limit of detection of the LC-MS/MS method. A peak eluting at approximately 3.7 min was observed in the SN-38 G channel for both dose treatments, however a peak of similar intensity and retention time was also observed for the DMSO control treated cells (Figure SI-5 A) indicating that it was not a SN-38 G metabolite. Further analysis of the 3.7 min peak by LC-MS/MS showed that no product ions related to SN-38 G were detectable in the product ion spectrum and alterations of the initial gradient conditions did not affect the retention time of the peak indicating that it was a polar contaminant present in the DMSO. Similar LC-MS/MS SRM ions chromatograms were obtained for PBLs from a second donor treated with 5 μM SN-38 for 6 and 24 h (Figures SI-6 A and SI-6 B). A peak corresponding to SN-38 was clearly detectable for both time points but no peaks were detected corresponding to the SN-38 G metabolite.

**Figure SI-5. Typical LC-MS/MS SRM ions chromatograms for PBL cell extracts from donor 1 treated with A) DMSO control B) 0.05 μM SN-38 for 4 hours and C) 5 μM SN-38 for 4 hours**.

Figure SI-6. Typical LC-MS/MS SRM ions chromatograms for PBLs cell extracts from donor 2 treated with 5 μM SN-38 for A) 6 hours and B) 24 hours

Overall, this metabolism data provides evidence that PBLs are not a good surrogate for predicting the effects of irinotecan. The LC-MS results demonstrated that the PBLs converted only a very small proportion of irinotecan to SN-38 and provided confirmation that the low ACA dose response of PBLs treated directly with irinotecan *ex vivo* was due to the inefficient production of SN-38.

There was no evidence for the occurrence of SN-38 glucuorinidation in PBLs *ex vivo*. The toxicity of irinotecan treatment is due to the slow metabolism, therefore the over-accumulation of SN-38 and the absence of glucuoronide metabolites may in part explain why the ACA results in the clinical studies did not correlate with irinotecan toxicities.
